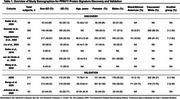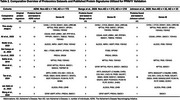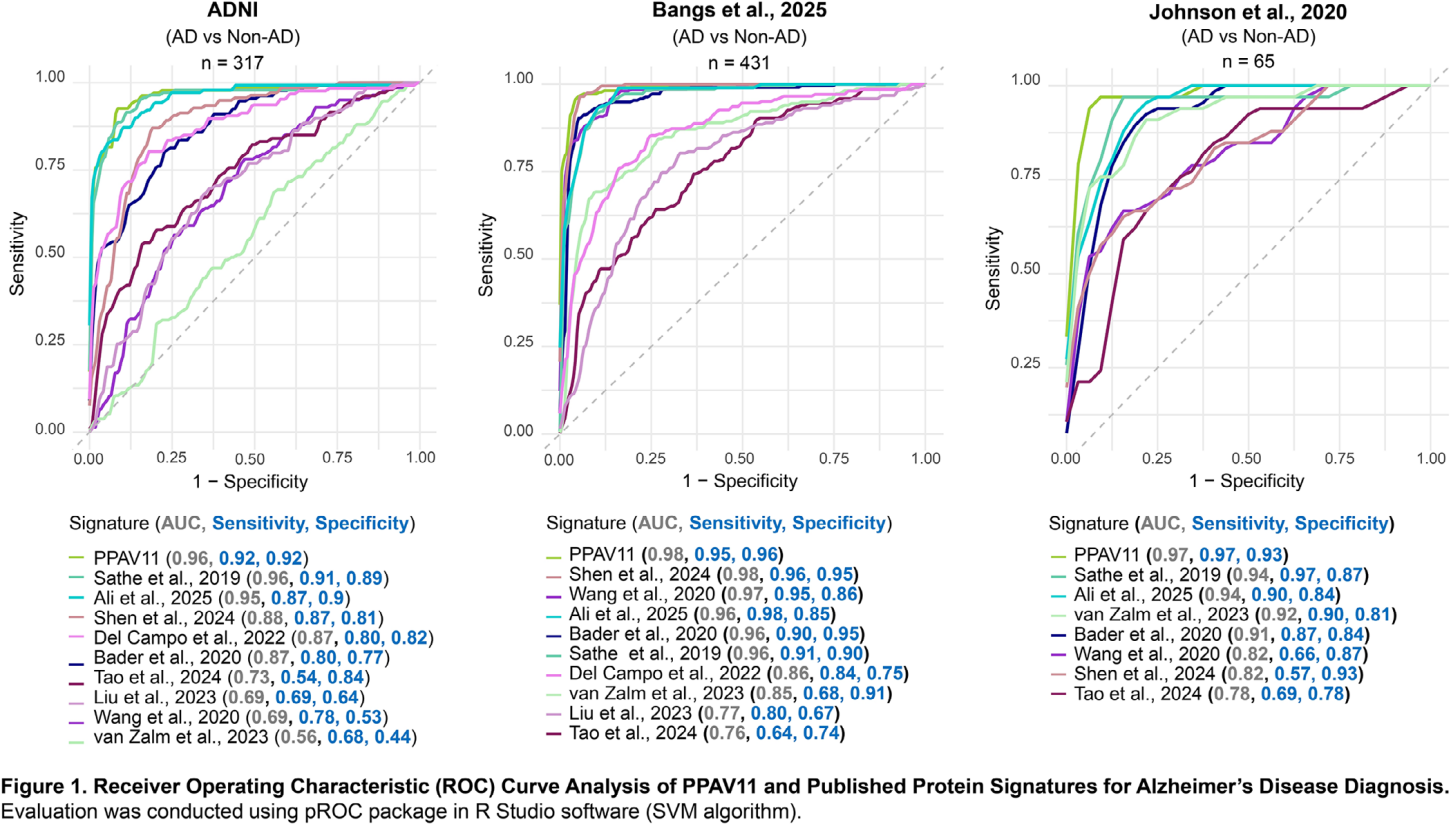# Identification of an Alzheimer’s Disease Biomarker Signature Using Reproducible Proteomics Data Mining

**DOI:** 10.1002/alz70861_108661

**Published:** 2025-12-23

**Authors:** María Fernanda Zambrano‐Astorga, Aldo Moreno‐Ulloa

**Affiliations:** ^1^ CICESE, Ensenada, BJ Mexico

## Abstract

**Background:**

Traditional diagnosis of Alzheimer’s disease (AD) has focused on detecting beta‐amyloid and phosphorylated tau, which only capture a portion of the disease’s complexity. Proteomic studies have revealed broader molecular and cellular alterations in AD, highlighting the need for novel biomarkers that provide a more comprehensive understanding of the disease. In this study, we performed multi‐cohort data mining of published proteomics datasets to identify a reproducible cerebrospinal fluid (CSF) biomarker signature for AD diagnosis.

**Method:**

We systematically selected seven CSF proteomics studies (n = 1,350) comparing AD patients to non‐AD subjects, using predefined inclusion and exclusion criteria. Dysregulated proteins were identified based on statistical significance (p < 0.05) and a fold change threshold (|log2 FC| ≥ 0.6). To ensure reproducibility, a co‐occurrence analysis was conducted to retain proteins dysregulated in at least two independent studies. The diagnostic performance of the resulting protein signature was evaluated using support vector machine‐based ROC curve analysis across three independent proteomics‐based cohorts (n = 813). We also compared the diagnostic performance of our signature with that of previously reported AD biomarker panels.

**Result:**

Across the seven studies included (Table 1), we identified 61 dysregulated proteins that overlapped between at least two datasets. After extensive filtering, 11 proteins demonstrating consistent directionality of change were consolidated into a reproducible diagnostic signature, referred to as PPAV11 (Table 2). This signature demonstrated outstanding diagnostic performance, with ROC‐AUC values ranging from 0.96 to 0.98 and sensitivity/specificity values > 0.92. Compared to nine previously published signatures, our panel consistently showed superior diagnostic accuracy across three independent proteomics datasets (Figure 1).

**Conclusion:**

This study identified a reproducible and robust 11‐protein CSF signature capable of accurately distinguishing individuals with AD from those without AD. Our biomarker panel outperformed several previously published signatures in diagnostic metrics. These findings highlight the potential of proteomics‐based approaches for improving disease diagnosis.

Data collection and sharing for ADNI cohort dataset was funded by the National Institutes of Health Grant U01 AG024904 and Department of Defense award number W81XWH‐12‐2‐0012.